# Assessing Caregiver Comfort With Linking the Health Care System and the Special Supplemental Nutrition Program for Women, Infants, and Children (WIC)

**DOI:** 10.2196/89731

**Published:** 2026-05-06

**Authors:** K Alexander Soltany, Kristina H Lewis, Beatriz Ospino-Sanchez, Angelina Pack, Kimberly Montez

**Affiliations:** 1 Office of Student Affairs, Wake Forest University School of Medicine, Winston-Salem, NC, United States; 2 Department of Epidemiology & Prevention, Wake Forest University School of Medicine, Winston-Salem, NC, United States; 3 Department of Pediatrics, Wake Forest University School of Medicine, One Medical Center Blvd, Winston-Salem, NC, 27157, United States, 1 336-716-2588

**Keywords:** WIC, food insecurity, electronic health records, Special Supplemental Nutrition Program for Women, Infants, and Children, data sharing

## Abstract

This study assessed families’ comfort levels with information-sharing between health care providers and the Special Supplemental Nutrition Program for Women, Infants, and Children (WIC) using a survey-based approach.

## Introduction

The Special Supplemental Nutrition Program for Women, Infants, and Children (WIC) is an evidence-based federal nutrition program that provides nutrition education, breastfeeding support, health care referrals, and access to healthy foods for low-income families [1]. Studies demonstrate that WIC participation is associated with improved birth outcomes, reduced infant mortality, and better overall child nutrition [2]. Given the benefits of WIC and underenrollment among eligible children, there is increasing interest in strengthening partnerships between health care settings and WIC to facilitate family enrollment and engagement [3].

Although health care systems and WIC care for a shared population, they exist in information silos, utilizing different electronic systems. While states allow sharing of specific data between health care settings and WIC, a form must be completed and faxed, creating barriers. Integrating WIC referrals within clinical care may improve WIC uptake, data sharing, and nutrition/health outcomes, but little research has explored family perspectives about information exchange between health care providers and WIC regarding their child’s health [4]. Better understanding family perceptions of information exchange could facilitate improvements in the design of effective, family-centered referral pathways. This study aims to assess families’ comfort levels with information-sharing between health care providers and WIC using a survey-based approach.

## Methods

### Study Design and Intervention

This cross-sectional study was part of a mixed methods study that implemented an innovative electronic health record (EHR)–based WIC screening and referral tool [5,6]. Briefly, WIC staff in three counties were granted secure, online, read-only access to the health system’s EHR [7], allowing review of medical charts, sending and receiving secure messages with health care teams, and receiving WIC referrals. This intervention was permissible under a memorandum of understanding that allowed data sharing between the health care system and county health departments. Participants gave verbal permission to have their information shared with WIC, which was documented in the EHR.

### Data Collection and Analysis

To understand parent/caregiver perceptions of information-sharing, a telephone-based survey was developed based on a review of the literature [8]. The survey was pilot-tested for face validity with resulting minor changes in sentence structure or word choice. The survey had four sections: (1) WIC participation history, (2) WIC benefit usage, (3) communication between health care team and WIC, and (4) self-reported demographics, including race/ethnicity (Hispanic vs non-Hispanic and Asian, Black, White, or Other [specify]) and food insecurity status using the Hunger Vital Sign [9]. Race/ethnicity were included as variables since WIC participation rates vary by race/ethnicity. Caregivers were eligible to participate if their child was ≤5 years old and attended one of eight participating clinics, received WIC benefits within the past year, underwent WIC EHR screening, and spoke English/Spanish. Eligible participants were purposively sampled via weekly EHR extractions of WIC screening results and were recruited via telephone, consented, and verbally administered the survey by trained research staff (AP, BO-S) in the participant’s preferred language. The staff member conducting the survey in Spanish was certified bilingual. Recruitment occurred from March to July 2022. Results were entered into Research Electronic Data Capture. Participants received a US $25 gift card as renumeration.

Results were analyzed using descriptive statistics. Bivariate comparisons between participant groups based upon demographic and other characteristics were made using *χ*^2^ testing or *t* testing (SAS, version 9.4; SAS Institute).

### Ethical Considerations

The Wake Forest University School of Medicine Institutional Review Board approved this study (IRB00078127).

## Results

Of 100 survey respondents, 93% were mothers. Respondents had a mean age of 29.9 years; 33% were Black, 19% White, and 53% Hispanic; 53% had completed high school (Table 1).

**Table 1.** Participant demographics (N=100).

Participant demographics	Values
Caregiver relationship, n (%)	
Mother	93 (93)
Father	4 (4)
Nonrelated caregiver	3 (3)
Caregiver age, mean (SD)	29.9 (6.1)
Caregiver race, n (%)	
White	19 (19)
Black	33 (33)
Other[Table-fn T1_FN1]	48 (48)
Caregiver ethnicity, n (%)	
Hispanic	53 (53)
Non-Hispanic	45 (45)
Missing	2 (2)
Child age, mean (SD)	3.5 (0.98)

a Other race/ethnicity included: Costa Rican, Hispanic, Mexican, Mayan, multiracial, Puerto Rican, and Salvadorian. These answers were provided via an open-ended text box after “Other” was chosen.

One third (35%) reported food insecurity (FI); 90% reported receiving WIC benefits for ≥1 year (see Multimedia Appendix 1 for full survey results). Overall, 79% were comfortable and 17% were uncomfortable about their child’s doctor communicating directly with WIC. Ninety-one percent were very or somewhat comfortable with WIC accessing their child’s EHR and 6% were uncomfortable; 95% were very or somewhat comfortable with secure messaging between their child’s doctor and WIC. Caregiver race/ethnicity and age were not associated with reported comfort levels (Table 2).

**Table 2.** Bivariate comparison between demographics and comfort levels with different activities involving the Special Supplemental Nutrition Program for Women, Infants, and Children’s (WIC’s) access to electronic health record (EHR) data.

Demographic	Comfortable with doctor communication with WIC^a^	*P* value	Comfortable with WIC staff having access to child’s EHR^a^	*P* value	Comfortable with doctors and WIC using the EHR to send secure messages^a^	*P* value
Caregiver race, %						
White	79.0	.84^b^	89.5	.96^b^	89.5	.21^b^
Black	78.8		90.9		100	
Other	83.3		91.7		93.8	
Caregiver ethnicity, %						
Hispanic (n=53)	84.9	.52^b^	92.5	.81^b^	94.3	.39^b^
Non-Hispanic (n=45)	80.0		91.1		97.8	
Comparing mean (SD) age (years) across comfortable versus uncomfortable caregivers						
Comfortable**^a^** caregivers	29.7 (6.3)	.57^c^	30.0 (6.1)	.60^c^	29.9 (6.2)	.79^c^
Uncomfortable caregivers	30.6 (5.3)		28.9 (6.6)		30.6 (4.0)	

a For the “Doctor Communication” question, “comfortable” designation combines individuals with “comfortable” and “neither comfortable nor uncomfortable” survey responses, and for the EHR questions, the “comfortable” designation combines individuals with “very comfortable” and “somewhat comfortable” survey responses in Multimedia Appendix 1.

b For comparing rates of “comfortable” responses across categorical groups (eg, race, ethnicity), *P* values are for *χ*2 tests.

c To compare the mean ages of caregivers who were comfortable versus not for each task, *t* tests were used.

## Discussion

While pediatricians, WIC nutritionists, and caregivers alike have identified data security and confidentiality as potential barriers to implementing integrated care models [4], WIC participants in this study were overwhelmingly comfortable with information sharing and integrated communication between health care providers and WIC. These comfort levels did not significantly vary by caregiver age, race, or ethnicity, signifying broad acceptability across demographic groups. Over one-third of caregivers reported FI, and almost all received WIC benefits for a year or longer.

These findings highlight an opportunity—caregivers were largely open to enhanced communication between health care systems and WIC. A prior study showed the majority of health care providers and WIC staff in favor of an EHR-based referral intervention [5]. Given the high prevalence of FI and the favorable attitudes toward EHR-based information sharing, health care systems may be uniquely positioned to bridge service gaps through continued thoughtful collaboration with community-based nutrition programs like WIC. Increasing the uptake of WIC and enhancing communication between the two systems have public health relevance, such as strengthening prevention/early intervention efforts on FI and obesity and reducing inconsistent messaging [10].

Although the American Academy of Pediatrics and the National WIC Association recommend health care referrals to WIC [11,12], practical and policy barriers prevent uptake of this recommendation. Chief among these policy barriers is that, without specialized legal data sharing agreements, bidirectional data sharing between health care systems and WIC is prevented by the Health Insurance Portability and Accountability Act [13]. Modifying such policies to allow for easier bidirectional data sharing could support direct referrals to/from health care practitioners and WIC, improve care coordination, and increase communication between the two entities, which often share populations but are siloed. To support such policies, more research is needed to determine whether WIC accessing the EHR improves efficiency of care and decreases costs and care duplication.

This study had several limitations. While modest, the sample size was limited by funding. Results are from one institution, which may limit generalizability of findings. Social desirability and selection biases may have influenced participant responses; all surveyed caregivers had consented to information sharing/referral, which may have prejudiced their comfort levels with bidirectional communication. Future studies should explore the impact of such information sharing on FI outcomes, parent/caregiver trust, and health care utilization and outcomes over time.

## Supplementary material

10.2196/89731 Multimedia Appendix 1 Survey responses.

## Abbreviations:

EHR: electronic health record
FI: food insecurity
WIC: Special Supplemental Nutrition Program for Women, Infants, and Children

## References

[1] (2025). WIC benefits and services. USDA food and nutrition service.

[2] Venkataramani M, Ogunwole SM, Caulfield LE (2022). Maternal, infant, and child health outcomes associated with the special supplemental nutrition program for women, infants, and children: a systematic review. Ann Intern Med.

[3] Davis RA, Leavitt HB, Chau M (2022). A review of interventions to increase WIC enrollment and participation. J Community Health.

[4] Bailey-Davis L, Kling SMR, Cochran WJ (2018). Integrating and coordinating care between the Women, Infants, and Children Program and pediatricians to improve patient-centered preventive care for healthy growth. Transl Behav Med.

[5] McCall A, Strahley AE, Martin-Fernandez KW (2024). WIC staff and healthcare professional perceptions of an EHR intervention to facilitate referrals to and improve communication and coordination with WIC: A qualitative study. J Clin Transl Sci.

[6] Monroe BS, Rengifo LM, Wingler MR (2023). Assessing and improving WIC enrollment in the primary care setting: a quality initiative. Pediatrics.

[7] (2023). Epic CommunityLink. Epic.

[8] (2001). National survey of WIC participants. https://fns-prod.azureedge.us/sites/default/files/WICSurvey.pdf.

[9] Hager ER, Quigg AM, Black MM (2010). Development and validity of a 2-item screen to identify families at risk for food insecurity. Pediatrics.

[10] Ali S, Gibbs SE, Wiseman K (2025). A qualitative study to understand parental, health care provider and WIC nutritionist perspectives on early childhood beverage choices for WIC-enrolled families in a Southeastern US health system. Matern Child Health J.

[11] Ashbrook A, Essel K, Montez K, Bennett-Tejes D (2021). Screen and intervene: a toolkit for pediatricians to address food insecurity. https://frac.org/wp-content/uploads/FRAC_AAP_Toolkit_2021_032122.pdf.

[12] (2024). Health care providers outreach toolkit. https://media.nwica.org/hcp%20toolkit%20-%20with%20how%20to%20establish%20referral%20and%20data%20sharing%20process.pdf.

[13] (2025). Summary of the HIPAA privacy rule. United States Department of Health and Human Services.

