# Supplementary material for: Assessing Caregiver Comfort With Linking the Health Care System and the Special Supplemental Nutrition Program for Women, Infants, and Children (WIC)
Source: JMIR Pediatr Parent. 2026 May 6;9:e89731. doi: 10.2196/89731 (PMC13148585; doi:10.2196/89731)
Supplement: Multimedia Appendix 1 [file pediatrics-v9-e89731-s001.docx]

**Table S1: Survey Responses**

| **Survey Topic** | **n/%** |
| --- | --- |
| **Length of WIC benefit receipt** |  |
| <1 year | 10 (10%) |
| 1-2 years | 26 (26%) |
| 3-5 years | 27 (27%) |
| >5 years | 30 (30%) |
| Can’t remember | 7 (7%) |
| **Help with signing up for WIC (yes – choose all that apply)** | 49 (49%) |
| Friend or family member | 14 (28%) |
| Doctor’s office | 27 (55%) |
| WIC program | 9 (18%) |
| Can’t remember | 0 (0%) |
| **How easy or hard signing up for WIC?** |  |
| Very easy | 91 (91%) |
| Somewhat easy | 6 (6%) |
| Somewhat hard | 2 (2%) |
| Can’t remember | 1 (1%) |
| **Why enroll in WIC? (choose all that apply)** |  |
| Help affording food for self | 50 (50%) |
| Help affording infant formula | 93 (93%) |
| Help affording infant/toddler food | 87 (87%) |
| Nutrition advice for self | 37 (37%) |
| Nutrition advice for child | 45 (45%) |
| Advice/help with breastfeeding | 35 (35%) |
| General health advice for self | 30 (35%) |
| General health advice for my child | 39 (39%) |
| **Describe participation since starting WIC** |  |
| Constantly enrolled since starting | 85 (85%) |
| Enrolled most of the time | 15 (15%) |
| **How often use WIC Electronic Benefit Card?** |  |
| Every day | 5 (5%) |
| At least once a week | 41 (41%) |
| A few times a month | 45 (45%) |
| Once a month or less | 9 (9%) |
| **How often run out of WIC benefits before end of month?**  *1 missing |  |
| Never | 28 (28%) |
| Rarely | 23 (23%) |
| Sometimes | 27 (27%) |
| Frequently | 11 (11%) |
| Always | 7 (7%) |
| Unsure | 3 (3%) |
| **How often meet with WIC nutritionist?** |  |
| Never | 2 (2%) |
| Twice a year or less | 3 (3%) |
| Every few months | 68 (68%) |
| Once a month | 27 (27%) |
| **WIC ever ask to obtain information from child’s doctor?** | 25 (25%) |
| **How easy or hard to obtain information from child’s doctor? (yes)** |  |
| Very easy to do | 19 (76%) |
| Somewhat easy to do | 5 (20%) |
| Somewhat hard to do | 1 (4%) |
| **Does child’s doctor communicate with WIC nutritionist?** |  |
| Yes | 5 (5%) |
| No | 27 (27%) |
| Unsure | 68 (68%) |
| **Comfort with child’s doctor communicating with WIC?** |  |
| Comfortable | 79 (79%) |
| Neither comfortable nor uncomfortable | 2 (2%) |
| Not comfortable | 17 (17%) |
| I don’t know | 2 (2%) |
| **Comfort with WIC access to child’s medical record?** |  |
| Very comfortable | 75 (75%) |
| Somewhat comfortable | 16 (16%) |
| Not at all comfortable | 6 (6%) |
| Unsure | 3 (3%) |
| **Comfort with WIC sending/receiving secure messages from child’s doctor?** |  |
| Very comfortable | 82 (82%) |
| Somewhat comfortable | 13 (13%) |
| Not at all comfortable | 2 (2%) |
| Unsure | 3 (3%) |
| **Household food insecurity last 12 months** |  |
| Often true | 9 (9%) |
| Sometimes true | 26 (26%) |
| Never true | 65 (65%) |
